# Fatty liver index is independently associated with deterioration of renal function during a 10-year period in healthy subjects

**DOI:** 10.1038/s41598-021-88025-w

**Published:** 2021-04-21

**Authors:** Satoko Takahashi, Marenao Tanaka, Masato Furuhashi, Norihito Moniwa, Masayuki Koyama, Yukimura Higashiura, Arata Osanami, Yufu Gocho, Hirofumi Ohnishi, Keita Numata, Takashi Hisasue, Nagisa Hanawa, Tetsuji Miura

**Affiliations:** 1 Department of Cardiovascular, Renal and Metabolic Medicine, Sapporo Medical University School of Medicine, S-1, W-16, Chuo-ku, Sapporo, 060-8543 Japan; 2 Department of Public Health, Sapporo Medical University School of Medicine, Sapporo, Japan; 3 Department of Health Checkup and Promotion, Keijinkai Maruyama Clinic, Sapporo, Japan

**Keywords:** Biomarkers, Nephrology, Hepatology

## Abstract

A potential link between chronic kidney disease (CKD) and non-alcoholic fatty liver disease (NAFLD) has been suggested. We investigated the relationship between fatty liver index (FLI), a noninvasive and simple predictor of NAFLD, and the development of CKD defined as estimated glomerular filtration rate < 60 mL/min/1.73 m^2^ or positive for urinary protein during a 10-year follow-up period in subjects who received annual health examinations (n = 28,890). After exclusion of CKD at baseline, a total of 14,163 subjects (male/female: 9077/5086) were recruited. During the 10-year period, 1458 males (16.1%) and 737 females (14.5%) had new onset of CKD. Multivariable Cox proportional hazard models with a restricted cubic spline showed that hazard ratios (HRs) of CKD development increased with a higher FLI at baseline in both males and females after adjustment of confounders. When divided by tertiles of FLI level at baseline (T1 ~ T3), the adjusted risk of CKD development in the T3 group (HR [95% confidence interval], male/female: 1.33 [1.16–1.54]/1.33 [1.08–1.63]) was significantly higher than that in both sexes in the T1 group as the reference. The addition of FLI into traditional risk factors significantly improved the discriminatory capability for predicting CKD. In conclusion, a high level of FLI predicts the development of CKD in both sexes in a general population.

## Introduction

Chronic kidney disease (CKD) has been recognized as a global public health problem^1^. Previous studies showed that about 10–15% of the adult population in developed countries suffers from CKD, and the prevalence of CKD is expected to increase further in the future due to increases in the elderly population and individuals with obesity and diabetes mellitus^2,3^. CKD not only precedes end-stage renal failure but also increases the risks of cardiovascular events and death even in early stages of CKD^1,4^. Therefore, comprehensive management of risk factors for CKD is clearly a current public need.

Non-alcoholic fatty liver disease (NAFLD) is associated with lifestyle-related diseases such as obesity and metabolic syndrome^5,6^. It has recently been proposed that liver disease associated with known metabolic dysfunction is newly defined as metabolic dysfunction-associated fatty liver disease (MAFLD)^7^. NAFLD has also been attracting attention as a cause of liver cirrhosis and hepatocellular carcinoma^8,9^. NAFLD is diagnosed in approximately 10–30% of adults by health examinations^10^, and the number of patients with NAFLD has recently been increasing^11^. It has been reported that NAFLD is a risk factor for insulin resistance, type 2 diabetes mellitus and cardiovascular disease^12,13^. Although diagnosis of NAFLD requires liver biopsy^14^, several noninvasive procedures have recently been established using liver ultrasonography, computed tomography, magnetic resonance spectroscopy and several biochemical indices including fatty liver index (FLI)^15^. FLI, which is calculated by using body mass index (BMI), waist circumference (WC) and levels of γ-glutamyl transferase (γ-GTP) and triglycerides, is a noninvasive and simple biomarker for diagnosis of NAFLD^16^ and has a high concordance with the histological criteria for NAFLD^17–19^. This noninvasive index enables analysis of the roles of NAFLD in various diseases using a large group of study subjects.

It has recently been reported that NAFLD diagnosed by liver biopsy, ultrasonography or altered serum liver enzymes is associated with the prevalence and incidence of CKD^20,21^. To the best of our knowledge, there have been only three studies on the association between FLI and CKD^22–24^. Cross-sectional studies showed that FLI level was associated with decreased estimated glomerular filtration rate (eGFR), increased urinary albumin excretion and prevalence of CKD^22,23^. A longitudinal study using 4761 Korean subjects (male/female: 1808/2953) also showed that the risk of cumulative incidence of CKD during a 10-year follow-up period was higher in the high FLI group (FLI ≥ 60), a group diagnosed as NAFLD, than in the low FLI group (FLI < 30), a group diagnosed as non-NAFLD^24^. However, despite the fact that there is a sex difference in the level of FLI^18^, there have been no study in which the relationship between FLI level and CKD divided by sex was investigated^22–24^. Furthermore, the possibility of racial difference in cutoff levels of FLI for diagnosis of NAFLD^17–19^ cannot be excluded, and it is not clear whether categorization of FLI using FLI ≥ 60 or FLI < 30 is optimal. Therefore, the association between FLI and CKD has not fully been characterized. Considering the uncertainties in earlier studies^22–24^, in the present study, we investigated the relationships of FLI value as a continuous variable and categorized tertile subgroups with the development of CKD during a 10-year follow-up period in a large number of subjects divided by sex.

## Results

### Characteristics of the study subjects

Basal characteristics of the recruited subjects are shown in Table 1. Components of FLI calculation, including BMI, WC, γ-GTP and triglycerides, were significantly higher in male subjects than in female subjects. FLI level was significantly higher in male subjects (median [interquartile ranges] 34 [16–58]) than in female subjects (7 [3–17]).

**Table 1:** Characteristics of the recruited subjects.

	Total	Male	Female	P
	n = 14,163	n = 9077	n = 5086	
Age (years)	47 ± 10	48 ± 10	46 ± 10	< 0.001
Body mass index	23 ± 3	24 ± 3	22 ± 3	< 0.001
Waist circumference (cm)	83 ± 9	86 ± 8	79 ± 9	< 0.001
Systolic blood pressure (mmHg)	116 ± 16	119 ± 16	110 ± 16	< 0.001
Diastolic blood pressure (mmHg)	74 ± 11	77 ± 11	69 ± 10	< 0.001
Smoking habit	4902 (36.2)	3983 (45.9)	919 (18.9)	< 0.001
Alcohol drinking habit	6423 (45.4)	5122 (56.4)	1301 (25.6)	< 0.001
**Comorbidity**				
Hypertension	2274 (16.1)	1760 (19.4)	514 (10.1)	< 0.001
Diabetes mellitus	657(4.6)	559 (6.2)	98 (1.9)	< 0.001
Dyslipidemia	3226 (22.8)	2003 (22.1)	1223 (24.0)	0.008
**Biochemical data**				
Hemoglobin (g/dL)	14.3 ± 1.5	15.1 ± 1.1	12.9 ± 1.2	< 0.001
Albumin (g/dL)	4.4 ± 0.2	4.4 ± 0.2	4.3 ± 0.2	< 0.001
Blood urea nitrogen (mg/dL)	14.0 ± 3.3	14.5 ± 3.2	13.1 ± 3.2	< 0.001
Creatinine (mg/dL)	0.72 ± 0.14	0.79 ± 0.10	0.59 ± 0.08	< 0.001
eGFR (mL/min/1.73 m^2^)	86.2 ± 13.9	85.1 ± 13.3	88.2 ± 14.8	< 0.001
Uric acid (mg/dL)	5.4 ± 1.4	6.0 ± 1.2	4.4 ± 0.9	< 0.001
AST (U/L)	21 (18–26)	22 (19–27)	19 (16–22)	< 0.001
ALT (U/L)	21 (15–31)	25 (18–36)	15 (12–20)	< 0.001
γ-GTP (U/L)	30 (19–55)	41 (27–72)	18 (14–27)	< 0.001
FPG (mg/dL)	93 ± 18	95 ± 20	87 ± 13	< 0.001
Hemoglobin A1c (%)	5.3 ± 0.7	5.4 ± 0.7	5.2 ± 0.5	< 0.001
LDL cholesterol (mg/dL)	121 ± 31	123 ± 31	117 ± 31	< 0.001
HDL cholesterol (mg/dL)	61 ± 16	56 ± 14	69 ± 15	< 0.001
Triglycerides (mg/dL)	91 (62–136)	109 (77–159)	66 (49–92)	< 0.001
FLI	22 (8–48)	34 (16–58)	7 (3–17)	< 0.001

Variables are expressed as number (%), means ± SD or medians (interquartile ranges).

*AST* aspartate aminotransferase, *ALT* alanine aminotransferase, *eGFR* estimated glomerular filtration rate, *FLI* fatty liver index, *FPG* fasting plasma glucose, *γ-GTP* γ-glutamyl transferase, *HDL* high-density lipoprotein, *LDL* low-density lipoprotein.

Basal characteristics of male and female subjects divided by subgroups according to tertiles of FLI at baseline are shown in Tables 2 and 3, respectively. Higher tertiles of FLI were accompanied by larger BMI and WC, higher frequencies of habits of alcohol drinking and smoking, hypertension, diabetes mellitus and dyslipidemia, higher levels of systolic and diastolic blood pressures, hemoglobin, platelet, uric acid, aspartate aminotransferase, alanine aminotransferase, γ-GTP, fasting plasma glucose, hemoglobin A1c, low-density lipoprotein (LDL) cholesterol and triglycerides and lower level of high-density lipoprotein (HDL) cholesterol in both sexes. In addition, higher tertiles of FLI were accompanied by higher blood urea nitrogen and lower eGFR in female subjects.

**Table 2:** Characteristics of male subjects divided by tertiles of FLI at baseline (n = 9077).

	T1 [0.8–21.9]	T2 [22.0–49.9]	T3 [50.0–99.7]	P
	n = 3072	n = 3002	n = 3003	
Age (years)	46 ± 11	49 ± 9	48 ± 9	< 0.001
Body mass index	21 ± 2	24 ± 2	26 ± 3	< 0.001
Waist circumference (cm)	79 ± 5	86 ± 5	93 ± 7	< 0.001
Systolic blood pressure (mmHg)	114 ± 15	119 ± 15	124 ± 15	< 0.001
Diastolic blood pressure (mmHg)	73 ± 10	77 ± 10	80 ± 10	< 0.001
Smoking habit	1,327 (45.2)	1,280 (44.5)	1376 (48.0)	0.021
Alcohol drinking habit	1,526 (49.7)	1,761 (58.7)	1835 (61.1)	< 0.001
**Comorbidity**				
Hypertension	300 (9.8)	587 (19.6)	873 (29.1)	< 0.001
Diabetes mellitus	103 (3.4)	188 (6.3)	268 (8.9)	< 0.001
Dyslipidemia	527 (17.2)	667 (22.2)	809 (26.9)	< 0.001
**Biochemical data**				
Hemoglobin (g/dL)	14.8 ± 1.0	15.1 ± 1.0	15.5 ± 1.0	< 0.001
Platelet (10^4^/μL)	23.0 ± 4.7	23.6 ± 4.9	23.6 ± 5.1	< 0.001
Albumin (g/dL)	4.4 ± 0.2	4.4 ± 0.2	4.4 ± 0.2	< 0.001
Blood urea nitrogen (mg/dL)	14.5 ± 3.4	14.6 ± 3.2	14.4 ± 3.1	0.043
Creatinine (mg/dL)	0.78 ± 0.10	0.79 ± 0.10	0.79 ± 0.10	< 0.001
eGFR (mL/min/1.73 m^2^)	86.8 ± 13.2	84.2 ± 13.2	84.3 ± 13.3	< 0.001
Uric acid (mg/dL)	5.6 ± 1.1	6.0 ± 1.2	6.5 ± 1.3	< 0.001
AST (U/L)	20 (17–23)	22 (19–26)	27 (22–34)	< 0.001
ALT (U/L)	18 (15–24)	25 (19–33)	37 (27–53)	< 0.001
γ-GTP (U/L)	25 (20–34)	42 (30–61)	77 (50–124)	< 0.001
FPG (mg/dL)	91 ± 18	95 ± 18	100 ± 23	< 0.001
Hemoglobin A1c (%)	5.2 ± 0.6	5.3 ± 0.7	5.5 ± 0.8	< 0.001
LDL cholesterol (mg/dL)	115 ± 28	126 ± 30	126 ± 32	< 0.001
HDL cholesterol (mg/dL)	62 ± 14	55 ± 13	51 ± 11	< 0.001
Triglycerides (mg/dL)	73 (57–94)	112 (88–145)	169 (126–233)	< 0.001

Variables are expressed as number (%), means ± SD or medians (interquartile ranges).

*AST* aspartate aminotransferase, *ALT* alanine aminotransferase, *eGFR* estimated glomerular filtration rate, *FLI* fatty liver index, *FPG* fasting plasma glucose, *γ-GTP* γ-glutamyl transferase, *HDL* high-density lipoprotein, *LDL* low-density lipoprotein.

**Table 3:** Characteristics of female subjects divided by tertiles of FLI at baseline (n = 5086).

	T1 [0.4–4.2]	T2 [4.3–12.0]	T3 [12.1–99.3]	P
	n = 1702	n = 1681	n = 1703	
Age (years)	41 ± 9	47 ± 10	51 ± 10	< 0.001
Body mass index	19 ± 2	21 ± 2	25 ± 3	< 0.001
Waist circumference (cm)	71 ± 5	78 ± 5	87 ± 8	< 0.001
Systolic blood pressure (mmHg)	104 ± 13	109 ± 15	117 ± 16	< 0.001
Diastolic blood pressure (mmHg)	65 ± 9	69 ± 10	74 ± 10	< 0.001
Smoking habit	294 (18.2)	288 (17.9)	337 (20.5)	0.123
Alcohol drinking habit	374 (22.0)	462 (27.5)	465 (27.3)	< 0.001
**Comorbidity**				
Hypertension	48 (2.8)	125 (7.4)	341 (20.0)	< 0.001
Diabetes mellitus	6 (0.4)	19 (1.1)	73 (4.3)	< 0.001
Dyslipidemia	334 (19.6)	385 (22.9)	504 (29.6)	< 0.001
**Biochemical data**				
Hemoglobin (g/dL)	12.7 ± 1.2	12.8 ± 1.2	13.2 ± 1.1	< 0.001
Platelet (10^4^/μL)	23.7 ± 5.0	24.6 ± 5.4	25.7 ± 5.7	< 0.001
Albumin (g/dL)	4.3 ± 0.2	4.3 ± 0.2	4.3 ± 0.2	0.065
Blood urea nitrogen (mg/dL)	12.9 ± 3.2	13.1 ± 3.1	13.4 ± 3.3	< 0.001
Creatinine (mg/dL)	0.59 ± 0.08	0.59 ± 0.08	0.58 ± 0.08	0.131
eGFR (mL/min/1.73 m^2^)	90.8 ± 14.7	87.3 ± 14.7	86.4 ± 14.6	< 0.001
Uric acid (mg/dL)	4.0 ± 0.8	4.3 ± 0.9	4.8 ± 1.0	< 0.001
AST (U/L)	18 (16–20)	18 (16–21)	20 (17–24)	< 0.001
ALT (U/L)	13 (11–16)	14 (12–18)	19 (15–27)	< 0.001
γ-GTP (U/L)	14 (12–18)	17 (14–23)	27 (19–42)	< 0.001
FPG (mg/dL)	84 ± 9	86 ± 12	92 ± 16	< 0.001
Hemoglobin A1c (%)	5.1 ± 0.4	5.2 ± 0.4	5.4 ± 0.6	< 0.001
LDL cholesterol (mg/dL)	105 ± 26	117 ± 29	130 ± 31	< 0.001
HDL cholesterol (mg/dL)	74 ± 14	71 ± 14	63 ± 15	< 0.001
Triglycerides (mg/dL)	48 (39–59)	67 (53–82)	98 (75–133)	< 0.001

Variables are expressed as number (%), means ± SD or medians (interquartile ranges).

*AST* aspartate aminotransferase, *ALT* alanine aminotransferase, *eGFR* estimated glomerular filtration rate, *FLI* fatty liver index, *FPG* fasting plasma glucose, *γ-GTP* γ-glutamyl transferase, *HDL* high-density lipoprotein, *LDL* low-density lipoprotein.

### Cumulative incidence of new onset of CKD during a follow-up period

The mean follow-up period was 6.3 years (range 1–10 years), and follow-up summation was 88,733 (male/female: 57,223/31,510) person-years. Among the 14,163 recruited subjects, 1458 male subjects (16.1%) and 737 female subjects (14.5%) had new onset of CKD during a 10-year period. Cumulative incidence of CKD was 21.1% (95% confidence intervals [CI] 20.2–21.9) (male/female 22.0% [95% CI 21.0–23.1]/19.3% [95% CI 17.9–20.6]).

### Impact of FLI level at baseline on development of CKD during a 10-year follow-up period

**Figure 1:**
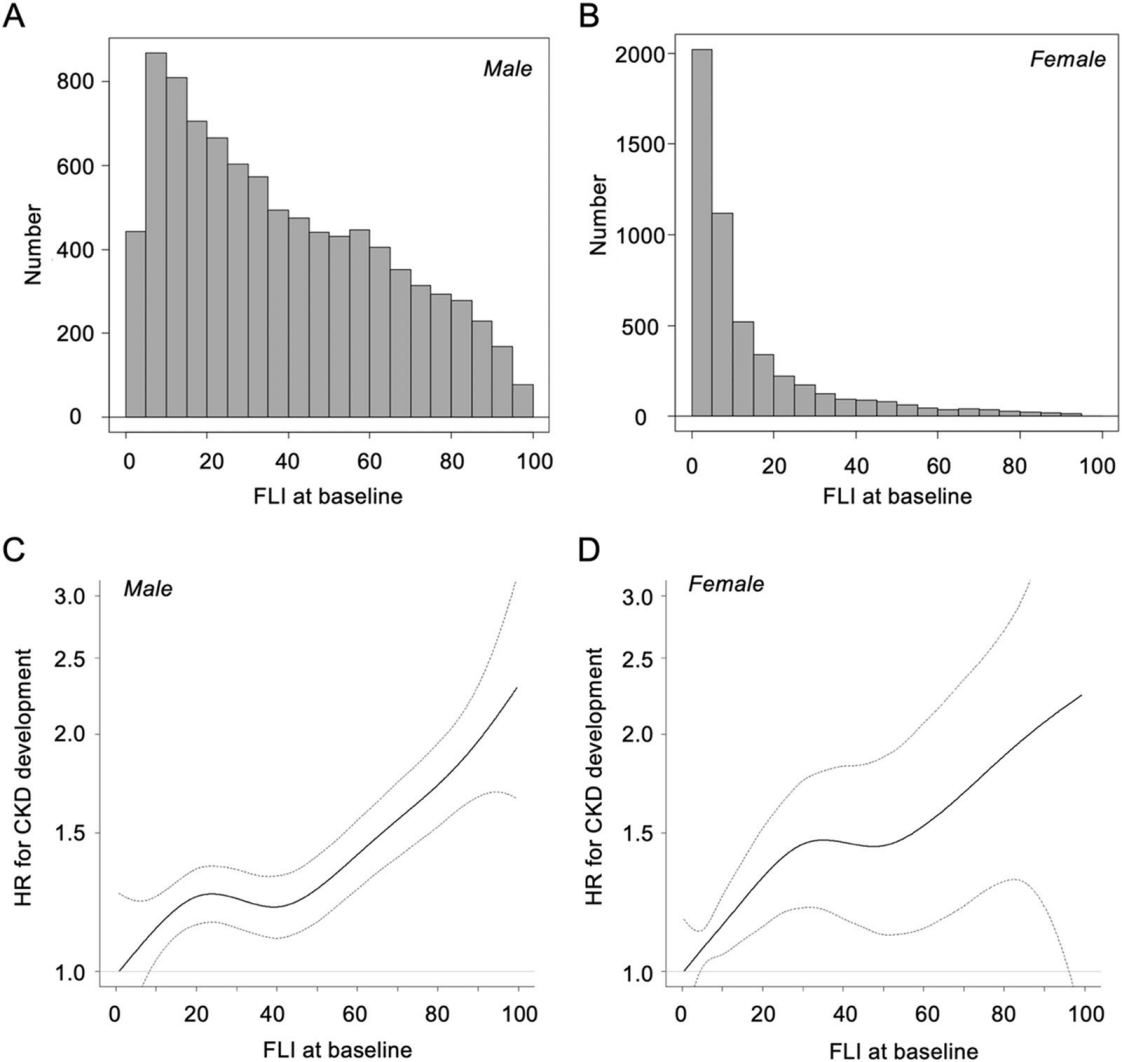
Hazard ratio of the development of CKD by FLI at baseline. (**A, B**) Histogram of fatty liver index (FLI) at baseline in male subjects (**A**) and female subjects (**B**). (**C,D**) Hazard ratios (HRs) for the development of chronic kidney disease (CKD) by FLI at baseline in male subjects (**C**) and female subjects (**D**) analyzed by multivariable Cox proportional hazard models with a restricted cubic spline after adjustment of age, estimated glomerular filtration rate, hemoglobin, uric acid, habits of smoking and alcohol drinking, and diagnosis of hypertension, diabetes mellitus and dyslipidemia at baseline. Solid line: HR; dashed line: 95% confidence interval. The reference values of FLI in male and female subjects were 0.8 and 0.4 as minimum values, respectively.

In all of the subjects, a multivariable Cox proportional hazard model analysis after adjustment of sex, age, eGFR, hemoglobin, uric acid, habits of smoking and alcohol drinking, and diagnosis of hypertension, diabetes mellitus and dyslipidemia at baseline showed that the adjusted hazard ratio (HR) for CKD development in the 3rd (T3) group of FLI (HR 1.31, 95% CI 1.16–1.47, P < 0.001) was significantly higher than that in the 1st tertile (T1) group of FLI as the reference (P for trend = 0.024) (Supplementary Table S1). There was no significant interaction between sex and tertiles of FLI for the development of CKD (P = 0.420). However, when subjects were divided by sex, the distribution patterns of FLI levels were different between males (Fig. 1A) and females (Fig. 1B).

In male subjects, a multivariable Cox proportional hazard model with a restricted cubic spline showed that the risk of CKD development increased with a higher FLI level at baseline after adjustment of age, eGFR, hemoglobin, uric acid, habits of smoking and alcohol drinking, and diagnosis of hypertension, diabetes mellitus and dyslipidemia at baseline (Fig. 1C). When the T1 group of FLI was used as the reference, multivariable Cox proportional hazard model analysis after adjustment of the covariates showed that HR for CKD development in the T3 group of FLI (HR 1.33, 95% CI 1.16–1.54, P < 0.001) was significantly higher than that in the T1 group of FLI in male subjects (P for trend < 0.001) (Table 4).

**Table 4:** Multivariable Cox proportional hazard analyses for new onset of CKD in tertiles of FLI.

	Male (n = 9077)				Female (n = 5086)			
	HR (95% CI)	P	HR (95% CI)	P	HR (95% CI)	P	HR (95% CI)	P
**FLI**								
T1	Reference	–	Reference	–	Reference	–	Reference	–
T2	1.14 (1.00–1.30)	0.058	1.01 (0.88–1.16)	0.900	1.16 (0.96–1.39)	0.120	1.15 (0.94–1.39)	0.170
T3	1.51 (1.33–1.71)	< 0.001	1.33 (1.16–1.54)	< 0.001	1.40 (1.18–1.68)	< 0.001	1.33 (1.08–1.63)	0.007
*P for trend*		< 0.001		< 0.001		< 0.001		0.007
Age (per 10 years)	–	–	1.04 (0.98–1.11)	0.190	–	–	0.82 (0.75–0.89)	< 0.001
eGFR (per 1 mL/min/1.73 m^2^)	–	–	0.96 (0.96–0.97)	< 0.001	–	–	0.97 (0.96–0.97)	< 0.001
Hemoglobin (per 1 g/dL)	–	–	0.99 (0.94–1.05)	0.780	–	–	0.91 (0.86–0.97)	0.005
Uric acid (per 1 mg/dL)	–	–	0.98 (0.93–1.02)	0.290	–	–	1.07 (0.98–1.16)	0.150
Smoking habit	–	–	1.24 (1.12–1.39)	< 0.001	–	–	1.22 (1.01–1.47)	0.042
Alcohol drinking habit	–	–	0.81 (0.73–0.90)	< 0.001	–	–	0.92 (0.77–1.09)	0.330
Hypertension	–	–	1.49 (1.32–1.70)	< 0.001	–	–	1.58 (1.25–1.98)	< 0.001
Diabetes mellitus	–	–	1.46 (1.20–1.77)	< 0.001	–	–	1.97 (1.28–3.02)	0.002
Dyslipidemia	–	–	1.00 (0.88–1.13)	0.990	–	–	1.15 (0.97–1.36)	0.100
	(AIC = 25,451)		(AIC = 23,989)		(AIC = 12,088)		(AIC = 11,374)	

*AIC* Akaike's information criterion, *CI* confidence interval, *CKD* chronic kidney disease, *eGFR* estimated glomerular filtration rate, *FLI* fatty liver index, *HR* hazard ratio.

In female subjects, a multivariable Cox proportional hazard model with a restricted cubic spline showed that the risk of CKD development after adjustment of age, eGFR, hemoglobin, uric acid, habits of smoking and alcohol drinking, and diagnosis of hypertension, diabetes mellitus and dyslipidemia at baseline increased with higher FLI at baseline (Fig. 1D). The HR after adjustment of the confounders in the T3 group of FLI (HR 1.33, 95% CI 1.08–1.63, P < 0.001) was significantly higher than that in the T1 group of FLI in female subjects (P for trend = 0.007) (Table 4).

### Discriminatory capacity of the addition of FLI for predicting the development of CKD

The addition of FLI level into traditional risk factors for CKD, including age, levels of eGFR, hemoglobin and uric acid, habits of smoking and alcohol drinking, and presence of hypertension, diabetes mellitus and dyslipidemia, modestly but significantly increased the area under the receiver operating characteristic curve in both males (0.657 vs. 0.662, P = 0.042) and females (0.645 vs. 0.651, P = 0.023) (Table 5). The incorporation of FLI level also led to significant improvements of the discriminatory capacity for predicting CKD development in the continuous net reclassification improvement and integrated discrimination improvement in both males and females (Table 5).

**Table 5:** Discrimination of the addition of FLI into traditional risk factors for CKD.

	AUC		NRI		IDI	
	Value (95% CI)	P	Value (95% CI)	P	Value (95% CI)	P
**Males**						
Traditional model^a^	0.657 (0.640–0.674)	–	–	–	–	–
Traditional model^a^ + FLI	0.662 (0.645–0.678)	0.042	0.135 (0.078–0.192)	< 0.001	0.004 (0.002–0.005)	< 0.001
**Females**						
Traditional model^a^	0.645 (0.621–0.668)	–	–	–	–	–
Traditional model^a^ + FLI	0.651 (0.628–0.675)	0.023	0.124 (0.047–0.201)	0.001	0.003 (0.001–0.005)	0.001

*AUC* area under the curve, *CI* confidence interval, *CKD* chronic kidney disease, *FLI* fatty liver index, *IDI* integrated discrimination improvement, *NRI* net reclassification improvement.

^a^Traditional model includes age, estimated glomerular filtration rate, hemoglobin, uric acid, smoking habit, alcohol drinking habit, hypertension, diabetes mellitus and dyslipidemia.

## Discussion

The present study showed that FLI level was independently associated with deterioration of renal function during a 10-year period in healthy subjects. Multivariable Cox proportional hazard models with a restricted cubic spline showed that HRs for CKD development after adjustment of traditional risk factors increased with a higher FLI level at baseline in both male and female subjects. In addition, HRs in the T3 group of FLI were significantly higher than those in the T1 group as the reference in both sexes. Furthermore, the addition of FLI into traditional risk factors significantly improved discriminatory capability of regression models for predicting CKD. Since there is a sex difference in data for components of FLI calculation, including BMI, WC, triglycerides and γ-GTP, being higher in male subjects than in female subjects^25–28^, it is necessary to analyze the FLI value divided by sex. In fact, there was a significant sex difference in FLI level in the present study as well as in a previous study^18^. A longitudinal study using 4761 Korean subjects (male/female: 1808/2953) showed that the HR for development of CKD defined as eGFR < 60 mL/min/1.73 m^2^ during a 10-year follow-up in the NAFLD group (FLI ≥ 60) was 1.5-times higher than that in the non-NAFLD group (FLI < 30)^24^. However, the sex difference in FLI level was not taken into consideration in that study^24^. When FLI level is analyzed by both sexes together, the risk for development of CKD might be underestimated in female subjects. It was found in the present study was that both male and female subjects with a high FLI level have an increased risk for the development of CKD and that a high FLI level can predict the development of CKD.

It has been reported that the cutoff level of FLI for diagnosis of NAFLD seems to be lower in Asians than in Europeans: FLI ≥ 30 in China^19^ and FLI ≥ 60 in Italy^16^. The subjects were not divided by sex in both of those studies, suggesting that there might be racial and sex differences in cutoff levels of FLI for diagnosis of NAFLD. To the best of our knowledge, the sex difference in the FLI value for diagnosis of NAFLD was considered in only one study performed in Taiwan, and the cutoff levels in male and female subjects in that study were FLI ≥ 35 and FLI ≥ 25, respectively^18^. In the present study, most of male (100%) and female (51%) subjects in the T3 group of FLI met the FLI criteria for diagnosis of NAFLD (male/female: FLI ≥ 35/FLI ≥ 25)^18^. In female subjects, FLI level might be a risk factor for CKD development regardless of the presence of NAFLD.

There are several possible mechanisms of the link between NAFLD and CKD development. The two diseases share risk factors for metabolic syndrome including obesity, insulin resistance, dyslipidemia and chronic inflammation^29,30^. As other possible mechanisms, a steatotic and inflamed liver has been reported to be a relevant source of proinflammatory, pro-fibrogenic and anti-fibrinolytic molecules including fetuin-A, fibroblast growth factor 2, tumor necrosis factor-α, transforming growth factor-β and plasminogen activator inhibitor-1, which theoretically can promote kidney injury^31^. Furthermore, fatty liver may promote glomerular injury and mesangial cell proliferation through increased secretion of very low-density lipoprotein and induction of atherogenic dyslipidemia such as triglycerides-rich lipoproteins and oxidized LDLs^31^. Xanthine oxidoreductase (XOR), a rate-limiting enzyme of uric acid production in the purine metabolism, is abundantly expressed in the liver and can increase reactive oxygen species by generating superoxide and hydrogen peroxide^32^. It has been reported that plasma activity of XOR is a novel biomarker of metabolic disorder^33^ and that change in XOR activity is significantly associated with changes in liver enzymes and body weight^34^. Inadequate activation of XOR in NAFLD may promote oxidative stress-related tissue injury including injury of the kidney^32^.

BMI, WC and triglycerides, components of FLI calculation, have been reported to be risk factors for the development of CKD^35,36^. The other component of FLI, γ-GTP, also known as γ-glutamyltransferase, is a cell-surface enzyme and has a physiological role in metabolizing extracellular reduced glutathione, a main antioxidant in mammalian cells^37^. Previous studies showed that elevated γ-GTP level was a significant predictor of hypertension, diabetes mellitus, cardiovascular disease, congestive heart failure and metabolic syndrome^38–41^. It has also been reported that γ-GTP level is associated with incidence of CKD in Asian subjects^38,42^, though a meta-analysis showed no significant association between elevated serum γ-GTP and risk of CKD in an adult general population^43^. Since FLI is an index that includes elements of metabolic syndrome^44^, FLI may represent integrated risk factors for CKD development.

Some interventional studies for improvement of renal function in patients with NAFLD have been carried out^45,46^. Modification of lifestyle improved liver histology and renal function in patients with non-alcoholic steatohepatitis^45^. Progression of CKD was also suppressed by reducing the waist-hip ratio in non-obese patients with NAFLD^46^. Reduction of visceral fat may affect functions of kidney and liver through a decrease in chronic low-grade systemic inflammation and suppression of the fibrotic process. Drug development and clinical trials for the treatment of NAFLD are currently being conducted worldwide^47^. Not only modification of conventional risk factors of CKD, which may be related to the common pathogenesis of CKD and NAFLD^29,30^, but also intervention for NAFLD by direct and indirect regulation of hepatokines and/or liver-derived molecules, in addition to modification of conventional CKD risk factors, might contribute to prevention of CKD. Reduction of the FLI level by treatment for NAFLD might be beneficial for prevention of CKD development. Further investigations are needed to determine whether FLI-guided prevention and/or treatment of NAFLD reduces the number of individuals with deterioration of renal function.

The present study has some limitations. First, since the study subjects had a yearly health check-up at a single urban clinic, the possibility of sample selection bias cannot be ruled out. Second, proteinuria was assessed only by the qualitative method since quantitative data for proteinuria were not available. Third, since diagnosis of hepatic steatosis was performed by FLI but not by imaging techniques, the severity of hepatic steatosis was not taken into consideration. Finally, since most of the participants in the present study were middle-aged subjects, the results of the present study may not be directly applicable to elderly subjects. Investigation of the relationship between FLI and CKD development in elderly subjects is needed in the future.

In conclusion, a high level of FLI, originally developed as an indicator of NAFLD, predicts new onset of CKD in both males and females. The addition of FLI into traditional risk factors significantly improves discriminatory capability for prediction of the development CKD. A further understanding of the mechanism of the link between FLI and CKD may enable the development of new therapeutic strategies for prevention of CKD.

## Methods

The present study was conducted as a project of the Broad-range Organization for REnal, Arterial and cardiac studies by Sapporo Medical University Affiliates (BOREAS) investigators and was designed as the BOREAS-CKD4 study. The study conformed to the principles outlined in the Declaration of Helsinki and was performed with the approval of the institutional ethical committee of Sapporo Medical University (Numbers: 29-2-64, 30-2-32). Written informed consent was obtained from all of the subjects.

### Study subjects

**Figure 2:**
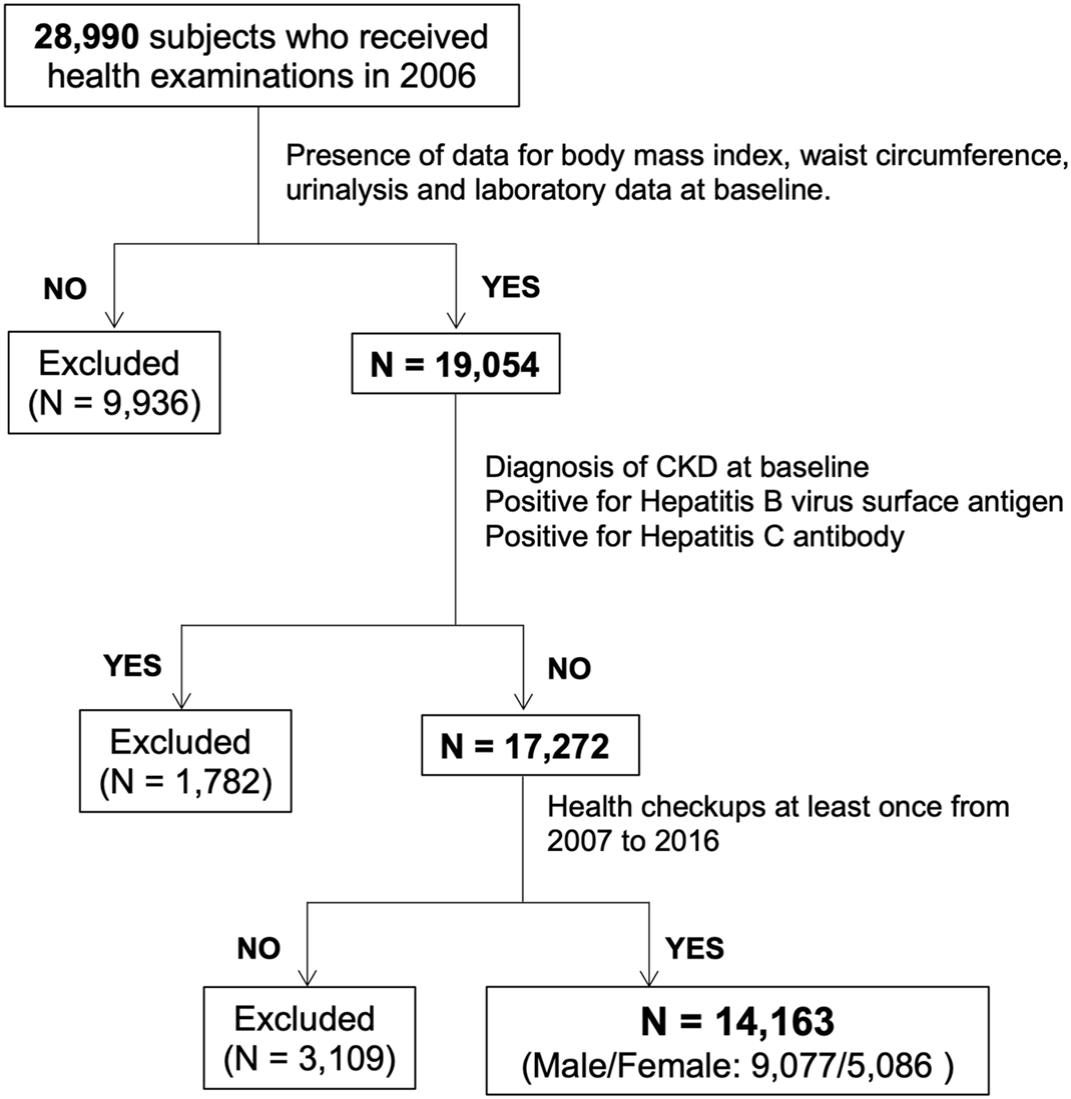
Flow chart of the selected study participants. Among 28,990 subjects enrolled in 2006, a total of 14,163 subjects (male/female: 9077/5086) were finally recruited for analyses in the present study. Chronic kidney disease (CKD) was defined as estimated glomerular filtration rate (eGFR) < 60 mL/min/1.73 m^2^ or positive for proteinuria.

All of subjects who received annual health examinations at Keijinkai Maruyama Clinic, Sapporo, Japan in 2006 were enrolled in this registry (n = 28,990)^48,49^. A flow chart of the study participants is shown in Fig. 2. Prespecified exclusion criteria were the absence of data for BMI, WC, urinalysis and laboratory data including serum creatinine, eGFR, triglycerides and γ-GTP at baseline, positive for hepatitis B virus surface antigen or hepatitis C virus antibody, and diagnosis of CKD at baseline. After exclusion, a total of 14,163 subjects (male/female: 9077/5086) who received health examinations at least once in the period from 2007 to 2016 were recruited in the present study. The number of subjects who received annual health checkup at the end of the follow-up period was 5683 (male/female: 3751/1932). A self-administered questionnaire survey was performed to obtain information on smoking habit, alcohol drinking habit and use of drugs for diabetes mellitus, hypertension and dyslipidemia.

### Measurements

Medical examinations, blood pressure measurements and samplings of urine and blood were performed after an overnight fast. Body height and weight were measured in light clothing without shoes, and BMI was calculated as body weight in kilograms divided by height in meters squared. As an indicator of renal function, eGFR was calculated by the following equation: eGFR (mL/min/1.73 m^2^) = 194 × serum creatinine^(−1.094)^ × age^(−0.287)^ × 0.739 (if female)^50^. FLI was calculated using the algorithm reported by Bedogni et al.^16^: FLI = [e^(0.953×ln(triglycerides)+0.139×BMI+0.718×ln(γ-GTP)+0.053×WC-15.745)^]/[1 + e^(0.953×ln(triglycerides)+0.139×BMI+0.718×ln(γ-GTP)+0.053×WC-15.745)^] × 100.

CKD was defined as eGFR < 60 mL/min/1.73 m^2^ or positive for urinary protein by the dipstick method. Diabetes mellitus was diagnosed in accordance with the guideline of the American Diabetes Association^51^: fasting plasma glucose ≥ 126 mg/dL, hemoglobin A1c ≥ 6.5% or self-reported use of anti-diabetic drugs. Hypertension was diagnosed as systolic blood pressure ≥ 140 mmHg, diastolic blood pressure ≥ 90 mmHg or self-reported use of anti-hypertensive drugs. Dyslipidemia was diagnosed as LDL cholesterol ≥ 140 mg/dL, HDL cholesterol < 40 mg/dL, triglycerides ≥ 150 mg/dL or self-reported use of anti-dyslipidemic drugs.

### Statistical analysis

Numeric variables are expressed as means ± SD for parameters with normal distributions and as medians (interquartile ranges) for parameters with skewed distributions. The distribution of each parameter was tested for its normality using the Shapiro–Wilk W test. Comparisons between two groups for parametric and nonparametric parameters were performed by using Student’s t-test and the Mann–Whitney U test, respectively. Clinical parameters were divided into three subgroups according to tertiles of FLI at baseline (T1 ~ T3) in both male and female subjects. Intergroup differences in percentages of demographic parameters were examined by the chi-square test. One-way analysis of variance for parametric parameters and the Kruskal–Wallis test for nonparametric parameters were used for detecting significant differences in data between multiple groups. The association between FLI levels at baseline and the development of CKD was investigated by multivariable Cox proportional hazard models with a restricted cubic spline after adjustment of confounders including age, eGFR, hemoglobin, uric acid, smoking habit, alcohol drinking habit and diagnosis of hypertension, diabetes mellitus and dyslipidemia at baseline. In addition, HRs and 95% CIs for the development of CKD in tertiles of FLI level at baseline were calculated by adjustment of the confounders. To compare the discrimination of CKD development between the models adjusted for confounders as traditional risk factors for CKD with and without FLI level, C-statistics analogous to the area under the receiver operating characteristic curve were estimated using the method of DeLong et al.^52^. Moreover, the increased discriminatory value of FLI level was examined by the continuous net reclassification improvement and integrated discrimination improvement^53^. A p value of less than 0.05 was considered statistically significant. All data were analyzed by using EZR^54^ and R version 3.6.1.

## Supplementary Information

Supplementary Table S1.

## Data Availability

The datasets analyzed during the current study are available from the corresponding author on reasonable request.
