# Supplementary material for: Fatty liver index is independently associated with deterioration of renal function during a 10-year period in healthy subjects
Source: Sci Rep. 2021 Apr 21;11:8606. doi: 10.1038/s41598-021-88025-w (PMC8060251; doi:10.1038/s41598-021-88025-w)
Supplement: Supplementary file 1 — Supplementary Table S1. [file 41598_2021_88025_MOESM1_ESM.pdf]

Supplementary materials

Fatty liver index is independently associated with deterioration of renal function during a 10-year period in healthy subjects.

Satoko Takahashi, Marenao Tanaka, Masato Furuhashi, Norihito Moniwa, Masayuki Koyama, Yukimura Higashiura, Arata Osanami, Yufu Gocho, Hirofumi Ohnishi, Keita Numata, Takashi Hisasue, Nagisa Hanawa, Tetsuji Miura.

Supplementary Table S1

Table S1. Multivariable Cox proportional hazard analyses for new onset of CKD in tertiles of FLI

	All (n = 14,163)			
	HR (95% CI)	P	HR (95% CI)	P
FLI				
T1	Reference	-	Reference	-
T2	1.15 (1.03-1.28)	0.012	1.05 (0.94-1.17)	0.422
T3	1.48 (1.33-1.64)	< 0.001	1.31 (1.16-1.47)	< 0.001
P for trend		< 0.001		0.024
Sex (Male)	-	-	1.09 (0.91-1.20)	0.522
Age (per 10 years)	-	-	0.94 (0.90-1.00)	0.466
eGFR (per 1 mL/min/1.73m^2)	-	-	0.96 (0.96-0.97)	< 0.001
Hemoglobin (per 1 g/dL)	-	-	0.99 (0.92-0.99)	0.026
Uric acid (per 1 mg/dL)	-	-	0.99 (0.95-1.03)	0.669
Smoking habit	-	-	1.23 (1.12-1.35)	< 0.001
Alcohol drinking habit	-	-	0.85 (0.78-0.93)	< 0.001
Hypertension	-	-	1.51 (1.35-1.68)	< 0.001
Diabetes mellitus	-	-	1.57 (1.31-1.88)	< 0.001
Dyslipidemia	-	-	1.05 (0.95-1.15)	0.347
	(AIC = 40,337)		(AIC = 38,069)	
Interaction: Sex-FLI tertiles				0.420

AIC, Akaike's information criterion; CI, confidence interval; CKD, chronic kidney disease; eGFR, estimated glomerular filtration rate; FLI, fatty liver index; HR, hazard ratio.
